# Novel Antarctic yeast adapts to cold by switching energy metabolism and increasing small RNA synthesis

**DOI:** 10.1038/s41396-021-01030-9

**Published:** 2021-07-22

**Authors:** D. Touchette, I. Altshuler, C. Gostinčar, P. Zalar, I. Raymond-Bouchard, J. Zajc, C. P. McKay, N. Gunde-Cimerman, L. G. Whyte

**Affiliations:** 1 Department of Natural Resource Sciences, McGill University, Sainte-Anne-de-Bellevue, QC Canada; 2 Department of Biology, Biotechnical Faculty, University of Ljubljana, Ljubljana, Slovenia; 3 Lars Bolund Institute of Regenerative Medicine, BGI-Qingdao, Qingdao, China; 4 Agricultural Institute of Slovenia, Ljubljana, Slovenia; 5 NASA Ames Research Center, Moffett Field, CA USA

**Keywords:** Soil microbiology, Fungal ecology, Microbial ecology

## Abstract

The novel extremophilic yeast *Rhodotorula frigidialcoholis*, formerly *R*. JG1b, was isolated from ice-cemented permafrost in University Valley (Antarctic), one of coldest and driest environments on Earth. Phenotypic and phylogenetic analyses classified *R. frigidialcoholis* as a novel species. To characterize its cold-adaptive strategies, we performed mRNA and sRNA transcriptomic analyses, phenotypic profiling, and assessed ethanol production at 0 and 23 °C. Downregulation of the ETC and citrate cycle genes, overexpression of fermentation and pentose phosphate pathways genes, growth without reduction of tetrazolium dye, and our discovery of ethanol production at 0 °C indicate that *R. frigidialcoholis* induces a metabolic switch from respiration to ethanol fermentation as adaptation in Antarctic permafrost. This is the first report of microbial ethanol fermentation utilized as the major energy pathway in response to cold and the coldest temperature reported for natural ethanol production. *R*. *frigidialcoholis* increased its diversity and abundance of sRNAs when grown at 0 versus 23 °C. This was consistent with increase in transcription of Dicer, a key protein for sRNA processing. Our results strongly imply that post-transcriptional regulation of gene expression and mRNA silencing may be a novel evolutionary fungal adaptation in the cryosphere.

## Introduction

The majority of the Earth’s biosphere exists at permanently cold temperatures, below 5 °C, and includes multiple cryoenvironments (<0 °C), many of which are characterized by some of the most dry, low biomass, cold, and salty conditions on Earth. Despite these harsh conditions, microorganisms capable of metabolic activity and even growth in situ have been reported in these habitats and include bacteria, archaea, algae, fungi [1–3]. These cold-adapted microorganisms are termed as psychrophilic (optimum temperature below 15 °C) or psychrotolerant (optimum above 15 °C) [4], and are able to maintain viability for thousands of years in glacial ice [5, 6]. Microbial communities in these environments have to overcome numerous biochemical and physiological challenges, including low water and nutrient availability, high oxidative stress, high solar irradiation, and multiple freeze-thaw cycles, coupled with a major decrease in membrane fluidity, enzymatic activity, and protein folding, and with the creation of stable secondary inhibitory DNA/RNA structures [4, 7–9].

While numerous studies have investigated bacteria, archaea and algae from Arctic and Antarctica environments [4, 10, 11], there has been a recent interest in fungi inhabiting these environments [12–14]. Many fungal species have been isolated and characterized from a diversity of extreme environments, such as brines [15], Arctic glaciers, and Antarctica rocks and deserts [5, 16, 17]. Fungi play key roles in the cryosphere as they are important facilitators of primary biomass production through endophytic and lichenic relationships [18, 19] and are involved in the nutrients recycling [20]. Although many basidiomycetous yeasts are adapted to low temperatures and detected in a broad range of cold ecosystems [21, 22], their adaptation strategies to low temperatures are not fully understood. Molecular mechanisms enabling yeast survival include the production of antifreeze and cold-active proteins, compatible solutes, and an increase in membrane fluidity [5, 23].

For example, the heterotrophic *Rhodotorula* yeasts are unicellular and pink-pigmented [24, 25] and have been isolated in many cold habitats [26]; numerous cold-adapted species, including *R. aurantiaca*, *R. psychrophila*, *R. psychrophenolica*, *R. glacialis*, and *R. himalayensis* [27, 28] have been identified. More recently, we isolated the putative novel psychrotolerant *Rhodotorula* JG1b strain from ~150,000-year-old ice-cemented permafrost soil from University Valley, in the McMurdo Dry Valleys of Antarctica [24], one of the coldest and driest places on Earth [29]. *Rhodotorula* JG1b was one of only six microorganisms isolated and sequenced form this ice-cemented permafrost. It was capable of growth at temperatures as low as −10 °C [1] and tolerated up to 15% NaCl and 12% perchlorate [24].

In this study, *Rhodotorula* JG1 was used as a model yeast to determine its adaptations to cold temperatures and how it survives in one of the coldest environments on Earth. Our objectives were to determine the phylogenetic position of *Rhodotorula* JG1b within the *Rhodotorula* genus and to identify and characterize its metabolic activity pathways and regulatory mechanisms in response to cold. To determine *R. frigidialcoholis’* response to colder temperatures, we characterized this strain’s metabolic capabilities at 0 °C, performed transcriptomic mRNAseq analysis in cultures grown at 0 and 23 °C, and assessed this species’ ethanol production capability at 0 °C compared to 23 °C. In addition, we analyzed the short non-coding RNAs to determine their role in post-transcriptional gene regulation to cold adaptation.

## Materials and methods

### Taxonomic analysis

*Rhodotorula* JG1b was isolated from ice-cemented permafrost soil in University Valley (Antarctica) (24). To determine the phylogenetic position of *Rhodotorula* JG1b, we performed phylogenetic, phenotypic, metabolic, and physiological characterization of this strain. Phylogenetic characterization and analyses were performed using the small and large subunits of the rRNA gene, the internal transcribed spacers (ITS) 1 and 2, translation elongation factor 1-α (TEF), and cytochrome b as well as publicly available whole genome sequences of the *Rhodotorula* genus (Supplementary Fig. S1). Detailed methodology of the phylogenetic analyses is described in the Supplementary material (Supplementary File 1 and Supplementary Fig. S2).

The morphology of the strain was determined by growth on potato dextrose agar (PDA), malt extract agar (MEA), cornmeal agar (CMA), and yeast extract peptone dextrose (YPD) [30], after incubation at 15 °C for 14 days. Morphological characters of pure cultures were observed on MEA with Nomarski interference contrast optics on an Olympus BX-51 microscope, colony color was described according to [31]. Growth at different temperatures (0, 4, 15, 20, 24, 30 and 37 °C) was determined in microtiter plates in liquid medium yeast nitrogen base. Three inocula were used (A, B, C) of optical density 0.4, 0.2, and 0.02, respectively, with 8 replicates/inocula. The growth curves were determined via OD at 590 nm. Assimilation of carbon and nitrogen sources were studied on three different sets of Phenotypic MicroArray (PM; Biolog) plates, PM1 MicroPlate and PM2A MicroPlate for carbon metabolism, and PM3B MicroPlate for nitrogen metabolism, as described by Viti et al., incubated at 20 °C for up to 14 days. Fermentation of D-glucose was tested in liquid medium with a 2% solution of sugar with Bromothymol blue and Durham tube [30], after an incubation of 12 days at 5, 15, 20, 30, and 37 °C.

### Culturing and growth conditions

For all subsequent experiments, *Rhodotorula* JG1b was cultured on PDB agar (HIMEDIA, Shenzhen, China). Biological triplicates of *Rhodotorula* JG1b were grown in 50 ml liquid cultures to early exponential phase in PDB at 0 °C without shaking, and at 23 °C with shaking at 150 RPM. Growth was monitored using OD_600_ and converted to viable cell numbers/ml based on CFU counts. Lack of shaking was necessary for the 0 °C treatment to allow growth at this low temperature since growth was not observed under shaking conditions at 0 °C. We separately ensured that the 0 °C non-shaking cultures remained aerobic (11.5 ± 0.98 mg O_2_/l) during growth by monitoring O_2_ concentrations using a Piccolo_2_ Fiber-Optic Oxygen Meter (Pyroscience, Aachen, Germany). O_2_ was not limited in this treatment, due to small culturing volume, slower growth rate at low temperature, and increase of oxygen solubility at lower temperature [32].

### Phenotypic MicroArray analysis

To physiologically characterize *Rhodotorula* JG1b metabolic capabilities under cold conditions, its assimilation of different carbon and nitrogen sources at 0 °C was assessed using the Biolog Phenotypic MicroArray (PM) technology (Biolog, Hayward, CA, United States). PM1 MicroPlate and PM2A MicroPlate were used for determining carbon utilization, and PM3B MicroPlate was used for nitrogen utilization. The plates were inoculated following Viti et al. protocol [33] with the following modifications: the initial inoculum was adjusted to OD_600_ 0.200, and the Biolog Dye Mix G was used. The duplicate plates were incubated at 0 °C, and at room temperature of 23 °C for comparison. Absorbance readings (590 nm) were taken at days 0, 1, 2, 6, 9, 14, 21, 28, 42, 56, 70, and 91 of incubation on a SpectraMax M2e microplate reader (Molecular Devices, San Jose, CA, United States). The measurements were normalized by the subtraction of both the *T*_0_ and the negative control. Absorbance values >0.2 were considered as positive values as previously described [34].

### RNA extraction, library preparation, and sequencing

To perform transcriptomic analysis, biological triplicate *Rhodotorula* JG1b cultures of each condition were grown as described in section “Culturing and growth conditions” to early exponential phase: OD_600_ 0.95 at 0 °C; and OD_600_ 6.20 at 23 °C. RNA was extracted using the Direct-zol RNA Miniprep Plus kit (#R2070) from Zymo Research (Irvine, CA, United States) on cultures with an initial resuspended pellet of approximately 10^8^ cells following the manufacturer’s instructions. An additional step of bead beating was also performed where resuspended cell suspensions were bead beat with 1:3 sterile glass beads (0.1 mm) for 45 s using the Mini-BeadBeater-24 (Biospec Products, Bartlesville, OK, United States) and centrifuged at 10,000 RCF for 2 min. Following a DNAse treatment with the TURBO DNAse kit (Invitrogen, Carlsbad, CA, United States), the RNA concentration was assessed with the Qubit 3.0 Fluorometer (Thermo Fisher Scientific, Waltham, MA, United States). RNA integrity was analyzed with the RNA Nano 6000 Assay Kit on the 2100 Bioanalyzer system (Agilent Technologies, Santa Clara, CA, United States). mRNA libraries were prepared following the TruSeq Stranded mRNA LT Sample Prep Kit (Illumina, San Diego, CA, United States) with an input material of 1.2 µg of total RNA. Libraries band sizes were confirmed on a 4% agarose gel.

We prepared small RNA (sRNA) (>200 nt) libraries for sequencing since we noted higher prevalence of sRNAs in the 0 °C cultures compared to the 23 °C cultures (Supplementary Fig. S3). sRNA libraries were prepared following the NEBNext Multiplex Small RNALibrary Prep Workflow (New England Biolabs, Ipswich, MA, United States). The cDNA constructs were purified with the Monarch PCR and DNA kit from New England Biolabs. Libraries band sizes were assessed with the Agilent High Sensitivity DNA kit on the 2100 Bioanalyzer system. Library bands from 105 to 210 bp were selected using the Pippin Prep 3% Agarose Gel Cassette from Sage Science (Beverly, MA, United States). mRNA sequencing was performed by RNASeq on the MiSeq system (Illumina) using the Reagent Kit v2 50 cycles and a 1 × 55 bp single-end configuration. sRNA sequencing was performed by RNASeq on the MiSeq system (Illumina) using the Reagent Kit v2 300 cycles and a 2 × 151 bp paired-end configuration. mRNA and sRNA raw sequence files (fastq) were uploaded to GenBank with accession id: PRJNA631292

### Bioinformatics

The mRNA raw sequencing reads were quasi-mapped and quantified with Salmon software [35] to the genome of *Rhodotorula* JG1b with the GenBank number GCA_001541205.1, and JGI IMG Genome ID 276120717 [24]. The analysis of the resulting quantifications was performed in R using the package DESeq2 [36]. Low count genes with less than 10 reads were removed before normalization of the samples. Hierarchical clustering and principal component analyses were used to check if the samples within the treatment (biological replicates) were more similar than between the treatments. The *p* value threshold for differential expression was 0.05. Log2 fold-change threshold was not used, but log_2_ fold-change shrinking was used for data visualization and gene ranking. Genes with a log_2_ fold-change (log_2_FC) ratio ≥∣1.5∣, and a *p* value <0.05 were considered as differentially expressed between the two growth conditions. Predicted protein KEGG assignments for each differentially expressed gene were downloaded from BlastKOALA website [37], to obtain function and metabolic pathway assignments. Heatmaps of the relative abundance of transcript expression of specific pathways were created. For those heatmaps, relative gene abundances of each sample were calculated by taking gene abundance (corrected on sequencing depth and length) and subtracting the average abundance across all samples.

sRNA raw sequence data were trimmed for adapter sequences and quantified using the CLC Genomics Workbench version 12 (https://www.qiagenbioinformatics.com/). The short rRNA fragments (rRFs)/microRNAs (miRNAs) sequences (15–30 nt) were extracted and counted from the total sRNA sequences using the CLC Genomics Workbench Small RNA Analysis tool. Identical short rRFs/miRNAs were grouped together and low count sequences with less than 50 reads were removed. The short rRFs/miRNAs were normalized based on sequencing coverage and to the ratio of sRNA/Total RNA, and differential expression was analyzed, with a *p* value threshold of 0.05. PCoA analysis (Bray–Curtis distance) was performed with the CLC Genomics Workbench version 12. The genomic origin of the ten highest expressed short rRFs/miRNAs were predicted with BLAST [38] by comparing to *Rhodotorula* genomes, and their potential mRNA targets were predicted using the RNAhybrid algorithm with the short rRFs/miRNAs sequences and all possible transcribed mRNA sequences as potential targets [39, 40]. The predicted protein mRNA targets of the short rRFs/miRNAs were identified with KEGG [37].

### Ethanol production assay

To corroborate the phenotypic and transcriptomic results, *Rhodotorula frigidialcoholis’* ethanol production capability was assessed. Triplicate *Rhodotorula* JG1b cultures were grown in identical media and conditions as with transcriptomic analysis, detailed in section “Culturing and growth conditions.” OD_600_ of the cultures was measured at regular time points for 83 days at 0 °C and for 17 h at 23 °C. Aliquots (1 ml) of the cultures at each time point were analyzed for ethanol production using the EnzyChrom Ethanol Assay Kit (BioAssay System, Hayward, CA, United States). Analysis of the ethanol production was done by comparing the trendline of ethanol concentration to the trendline of cell density over time. Ethanol concentrations were normalized to the ethanol evaporation rate that was experimentally determined using sterile PDB broth inoculated with 0.05% of ethanol.

## Results and discussion

### Taxonomy and description of *Rhodotorula frigidialcoholis* sp. nov

**Fig. 1:**
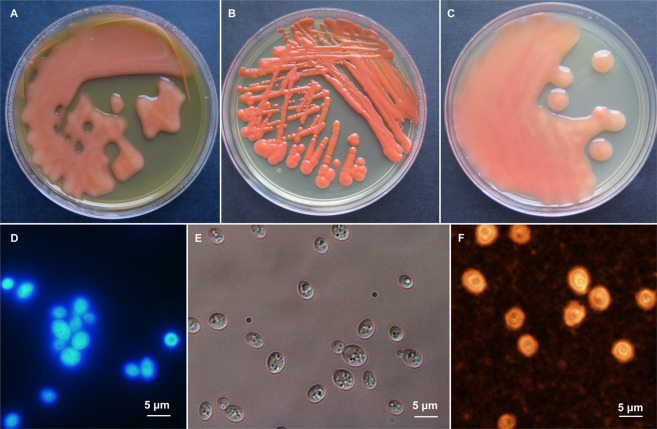
Morphology of *Rhodotorula frigidialcoholis*. **A**–**C** Cultures of *R. frigidialcoholis* on PDA (**A**), YPD (**B**), and MEA (**C**) in 9 cm Petri dishes after 2 weeks of incubation at 15 °C. Micromorphology of cells stained with calcofluor white viewed with fluorescent microscopy (**D**), mounted in water, viewed by DIC (**E**), stained with India ink, viewed with light microscopy (**F**), all after 2 weeks of growth at 15 °C.

The *Rhodotorula* yeast strain JG1b isolated from the upper-elevation McMurdo Dry Valleys of Antarctica [24] was determined to be a novel species based on phylogenetic and phenotypic analyses. Bayesian analysis of aligned, concatenated 18S, ITS, D1/D2 domains of 28S rRNA, and TEF sequences of species within the genus *Rhodotorula* resulted in a consensus tree shown in Supplementary Fig. S1A, while the phylogenetic tree based on available complete *Rhodotorula* genomes is shown in Supplementary Fig. S1B. According to these analyses, the JG1b strain forms a strongly supported monophyletic group within *Rhodotorula* species and is highly supported as the sister clade of *R. mucilaginosa* (bootstrap = 100%). Clustering analysis of the data obtained from 190 carbon and 95 nitrogen assimilation assays confirmed that *Rhodotorula* JG1b is distinct from other *Rhodotorula* species included in the Biolog phenotyping experiment (Supplementary Fig. S1C). Contrary to *R. mucilaginosa* and *R. alborubescens*, *Rhodotorula* JG1b is able to assimilate L-rhamnose, dulcitol, L-sorbose, and is unable to assimilate citrate and D-cellobiose (Supplementary Table S1). Based on the results presented here, we strongly suggest that *Rhodotorula* sp. JG1b is a novel species in the *Rhodotorula* genus. Accordingly, the species is taxonomically introduced herewith as *Rhodotorula frigidialcoholis* D. Touchette & P. Zalar, sp. nov. (frigidus in Latin meaning cold, alcohilis in Latin genitive case of alcohol; of cold alcohol, referring to ability of alcohol production in cold). Morphological characters of *R. frigidialcoholis* are shown in Fig. 1. Holotype for *Rhodotorula frigidialcoholis*designated herewith: EXF-10854H, permanently preserved and metabolically inactive deposit of the Culture Collection of Extremophilic Fungi Ex (EXF), University of Ljubljana, Slovenia. Ex-type cultures: EXF-10854 and CBS 16468 (Westerdijk Fungal Biodiversity Institute, Utrecht, The Netherlands). The strain EXF-10854 was isolated as JG1b from ~150,000-year-old ice-cemented permafrost of University Valley, the upper-elevation McMurdo Dry Valleys of Antarctica in 2009 by Jacqueline Goordial  (24). DNA sequence accession numbers derived from type: MT569975 (18S rRNA), MT569976 (D1/D2 of 28S rRNA), MT560678 (ITS), MT584855 (TEF1). MycoBank accession number for *Rhodotorula frigidialcoholis *is MB 835866. Diagnosis: After a 14-day incubation at 15 °C on 5% MEA agar, the streaked cultures are slimy, liquid and shinning with entire margin, greyish red (7B6) (Fig. 1). The cells are subglobose, 3.5 × 4.5 µm, and encapsulated, budding is unipolar (Fig. 1). Fermentation was not observed. Assimilated compounds are D-glucose, sucrose, raffinose, galactose, trehalose, maltose, melezitose, L-sorbose, L-rhamnose, D-xylose, Dl-arabinose, D-ribose, glycerol, galactitol, D-mannitol, and xylitol. Not observed assimilated compounds are inulin, melibiose, lactose, methyl A-D-glucoside, cellobiose, erythritol, myo-inositol, citrate, D-glucosamine, N-acetyl-D-glucosamine, nitrate, and nitrite. Hydrolysis of urea is positive. A complete list of assimilated and not assimilated compounds is listed in the Supplementary Table S1. Growth from 0 to 37°C (Supplementary Fig. S4).  Strain JG1b was previously described as able to grow as low as −10 °C [1, 24], but appears to have lost subzero growth capabilities through sub-culturing and long-term storage at −80 °C, as in this study *R. frigidialcoholis* was only successfully grown at 0 °C while incubated in a PDB 50 ml liquid culture, without shaking. An extended description of *Rhodotorula frigidialcoholis * is detailed in the Supplementary material (Supplementary file 1) a complete list of assimilated and not assimilated compounds in the Table S1.

### Phenotypic MicroArray response at cold temperatures

To characterize *R. frigidialcoholis* metabolic capabilities at low temperature, its substrate utilization was assessed through the Biolog Phenotypic MicroArray that determines the ability of a microorganism to utilize a variety of sole carbon or nitrogen sources [41]. Positive detection of metabolic activity relies on the transfer of electrons from the growth substrate, through the electron transport chain (ETC), to the tetrazolium redox dye, transforming the dye into a violet colored reduced form [41, 42]. After 91 days of incubation, *R. frigidialcoholis* utilized 35 on 190 substrates as a sole carbon source at 0 °C for growth (Supplementary Table S2). Only two (L-Proline, L-Pyroglutamate) of the 95 nitrogen sources were utilized as sole nitrogen sources at 0 °C after 91 days of incubation (Supplementary Table S2). Surprisingly, of the 35 positive carbon substrates, only six (d-Trehalose, d,l-α-Glycerol Phosphate, d-Mannose, Tween 20, Tween 40, and Tween 80) showed reduction of the dye and parallel evidence of microbial growth (Supplementary Table S2). This was puzzling because in the remaining 29 carbon sources, we observed active growth in the wells (based on OD_600_ and visual inspection) but without reduction of the dye; this growth was confirmed by streak plates. As the tetrazolium dye is reduced via the ETC [43], the observation of growth but no reduction of the dye suggest that at 0 °C *R. frigidialcoholis* potentially produces most of the energy for cellular growth through metabolic pathways other than ETC, such as glycolysis or perhaps fermentation. Damage to the mitochondrial DNA and the ETC in *S. cerevisiae* was previously shown to decrease tetrazolium dye reduction [44] while yeast mitochondria mutants have respiratory dysfunction and an inability to reduce tetrazolium salts [45–47]. Therefore, growth of *R. frigidialcoholis* coupled with a lack of tetrazolium dye reduction suggests that cell respiration through the mitochondria and ETC was significantly reduced while growth was still maintained at 0 °C.

### mRNA transcriptional responses to cold temperature

**Fig. 2:**
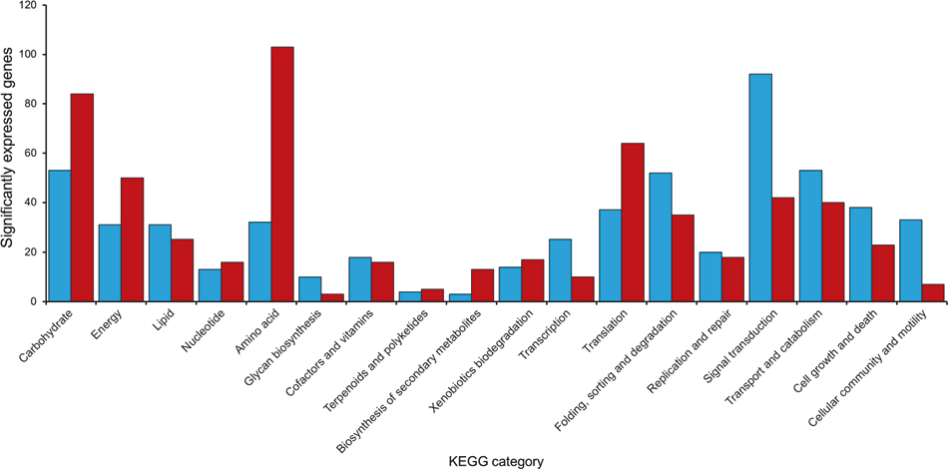
Number of gene significantly differentially expressed for each KEGG metabolic pathways and cellular processes in *Rhodotorula frigidialcoholis* at 0 °C compared to 23 °C. The total number of genes in each KEGG category that showed significant (*p* < 0.05) upregulation (≥1.5 log_2_FC) at 0 °C are indicated in blue. The total number of genes in each KEGG category that showed significant (*p* < 0.05) upregulation (≥1.5 log_2_FC) at 23 °C are indicated in red. Blue and red are equivalent to light gray and dark gray in the printed version.

To determine *R. frigidialcoholis* transcriptional response to cold, we performed comparative mRNA transcriptomic analysis on triplicate exponential phase cultures of *R. frigidialcoholis* grown at 0 and 23 °C. Overall, 1772 genes were significantly differentially expressed (*p* < 0.05) between these two conditions, including 994 genes overexpressed, and 778 genes downregulated at 0 °C compared to 23 °C (Supplementary Tables S3 and S4). Of these significantly differentially expressed genes, 52% were annotated with KEGG. The major global differences in transcript abundance between 0 and 23 °C were found in genes related to carbohydrate metabolism, energy metabolism, lipid metabolism and signal transduction, glycan biosynthesis/metabolism, transcription machinery, translation, and amino acid metabolism (Fig. 2). While protein levels are not always correlated with the transcript levels as they are also influenced by post-transcriptional, translational, and degradation regulation, mRNA levels do partially correlate with protein abundance [48] and are informative to deduce overall metabolic processes [49].

#### Cellular membrane and signal transduction

**Fig. 3:**
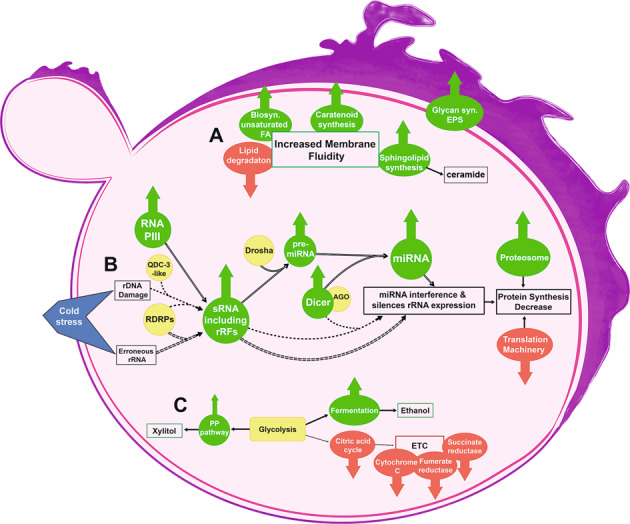
Diagram showing major processes and pathways increases during growth at 0 °C in *Rhodotorula frigidialcoholis*. **A** Cold induced modifications to *R. frigidialcoholis* membrane. **B** Potential cold induced RNAi mechanisms in *R. frigidialcoholis*. Hollow arrows represent the miRNA and short rRFs (miRNA-like) mechanisms [105, 107], dashed arrows represent the rRFs derived from rDNA damage mechanism [115], double-dashed arrows represent the rFRs derived from erroneous rRNA mechanism resulting in rRNA synthesis inhibition [114]. **C** Cold induced switch in *R. frigidialcoholis* metabolism. A list of the abbreviations is included in the Supplementary material (Supplementary Table S5).

Maintenance in membrane fluidity through lipid membrane component modification is one of the best-known adaption strategies in microorganisms living in cold environments [23]. Overall, *R. frigidialcoholis* overexpressed genes involved in lipid metabolism at 0 °C compared to 23 °C (Figs 2 and 3 and Supplementary Table S5). Similar to previous studies, genes related to unsaturated fatty-acids (FA) biosynthesis were overall overexpressed at 0 °C indicating increased unsaturated FA concentrations maintain membrane fluidity at cold temperatures [50–53]. Relatively high amounts of unsaturated FAs were also reported in other psychrophilic basidiomycetous yeasts isolated from Antarctic and Patagonian ecosystems [54–56].

Sphingolipid metabolism genes were also highly overexpressed at 0 °C compared to 23 °C in *R. frigidialcoholis* (Supplementary Fig. S4) presumably leading to increased biosynthesis of ceramide and phytoceramide. Phytoceramide synthesis genes were previously shown to be consistently up-regulated during low temperatures fermentation (12 °C) in *Saccharomyces cerevisiae* [57, 58]. Ceramide and other sphingolipid products can induce cell cycle arrest [59] indicating growth suppression in *R. frigidialcoholis* may be a response to freezing conditions in Antarctic permafrost habitats.

Multiple *Rhodotorula* species produce lipid antioxidants and photoprotective carotenoids [60–62], especially high concentrations of β-carotene, torulene, and torularhodin. Carotenoids help maintain membrane rigidity in cold-adapted microorganisms as they balance out the higher percentage of unsaturated FAs [50, 63]. At 0 °C, *R. frigidialcoholis* overexpressed the phytoene desaturase gene (FC = 4.46, *p* = 0.00) involved in the production of torulene and β-carotene precursors. This is corroborated by our observation of a darker pink color in *R. frigidialcoholis* grown at 0 °C compared to 23 °C. Carotenoids play an important role in UV and sunlight protection in yeast [61, 64, 65]. Thus, at 0 °C, *R. frigidialcoholis* may increase carotenoid biosynthesis both to regulate membrane fluidity and as an adaption to solar irradiation or long-term background γ radiation in the extreme University Valley permafrost environment.

The PI3k-Akt, Hippo, TGFß, VEGF, RAS, MAPK, RAP1, FoxO, and Wnt cell signal transduction pathways genes were overexpressed at 0 °C (Fig. 2 and Supplementary Table S4); these pathways are related to gene regulation, actin cytoskeleton formation, and cellular adhesion, and are activated by transmembrane receptors. While little is known about cold response signal transduction modulation, it has been linked to freezing temperature survival in plants [66, 67]. Perception of cold in *Rhodotorula* by cold-sensitive membrane receptors may also lead to a cascade response, thus preparing it for cold temperatures. In yeasts, mitogen activated protein kinase cascades play major roles in gene transcription and actin cytoskeletal organization [68]. Thus, *R. frigidialcoholis* may overexpress genes coding for the formation of the actin cytoskeleton (cdc42, actin δ/γ subunits) at 0 °C (Supplementary Table S4) as a way to transport lipids (biosynthesis of which is also overexpressed at 0 °C) to the membrane and maintain membrane fluidity (Figs 2 and 3 and Supplementary Fig. S4).

#### Differential expression of homologous proteins between 0 and 23 °C

We observed 19 differentially expressed homologous protein categories between *R. frigidialcoholis* cultures at 0 and 23 °C (Supplementary Table S6). Eight of these differentially expressed homologous protein categories were related to membrane structure and transport across membranes. For example, YAT, an amino acid transporter, had three homolog genes overexpressed at 0 °C, two homologs overexpressed at 23 °C, and two homologs that were not differentially expressed between the two temperatures (Supplementary Table S6). Homologous proteins arise from gene duplication and sometimes gain new functions [69]. Gene duplication, genome redundancy, and paralogous (homologs) genes increase the organism’s ability to grow under varied conditions (e.g., temperatures, substrates, pH, salinity) [2, 70, 71].

#### Glycan biosynthesis and metabolism

A significant increase in glycan biosynthesis genes was observed in *R. frigidialcoholis* at 0 °C compared to 23 °C (Figs 2 and 3). Glycans are carbohydrate-based polymers associated with cellular protection and storage [72] and are also important component of glycoproteins, including cell-surface membrane proteins, such as receptors and adhesion proteins [72, 73]. *Rhodotorula* species produce mannan, a mannose glycan extracellular polysaccharide (EPS) [74, 75]. Microbial EPS are components of microbial biofilms and constitute a protective matrix against the desiccation and environmental fluctuations [76, 77], including freeze-thaw damage [78]. At 0 °C, *R. frigidialcoholis* overexpressed genes involved in GDP-D-mannose synthesis (a glycan biosynthesis precursor), and overexpressed multiple genes coding for glycosyltransferase and glycosidases (Supplementary Table S4), which regulate glycans [79]. These results indicate that *R. frigidialcoholis* increases EPS synthesis at 0 °C through overexpression of mannan and other glycoproteins as adaptation to desiccation and freeze-thaw cycles of the Antarctic University Valley permafrost environment.

#### Transcription, translation, and amino acid metabolism

*R. frigidialcoholis* overexpressed multiple genes involved in the transcriptional machinery at 0 °C compared to 23 °C (Figs 2 and 3), including the overexpression of genes coding for the three RNA polymerase complexes (RNA Polymerase I, II, and III and the RNA polymerase II transcription factor) (Supplementary Table S4). A similar increase in the transcriptional machinery at 10 and 4 °C was reported in other yeasts [80–83]. Contrariwise, *R. frigidialcoholis* decreased expression of genes related to translation at 0 °C compared to 23 °C, such as the downregulation of genes involved in ribosomal protein synthesis and aminoacyl-tRNAs. In addition, *R. frigidialcoholis* overexpressed proteasome genes involved in protein degradation at 0 °C. Cold temperatures can reduce translation efficiency and protein synthesis by inducing formation of secondary structures in DNA/RNA molecules and by inactivating ribosomes [4, 83, 84]. While amino acid biosynthesis genes were overall downregulated at 0 °C compared to 23 °C (Fig. 2), the bacterium *Polaromonas* sp. also downregulated genes involved in amino acid biosynthesis and transport [8]. Thus, at low temperatures, *R. frigidialcoholis* may decrease amino acid synthesis and transport to slow down translation and focus energy on cold acclimation, in parallel with increased protein degradation to possibly recycle amino acids. A notable exception was of upregulation of the histidine pathway; this could be due to its role in membrane cold sensor histidine kinases [85] as was reported in the basidiomycetous yeast *Mrakia blollopis*, which accumulated aromatic amino acids, such as histidine, at −3 °C compared to 10 °C [86].

#### Carbohydrate metabolism and energy metabolism

**Fig. 4:**
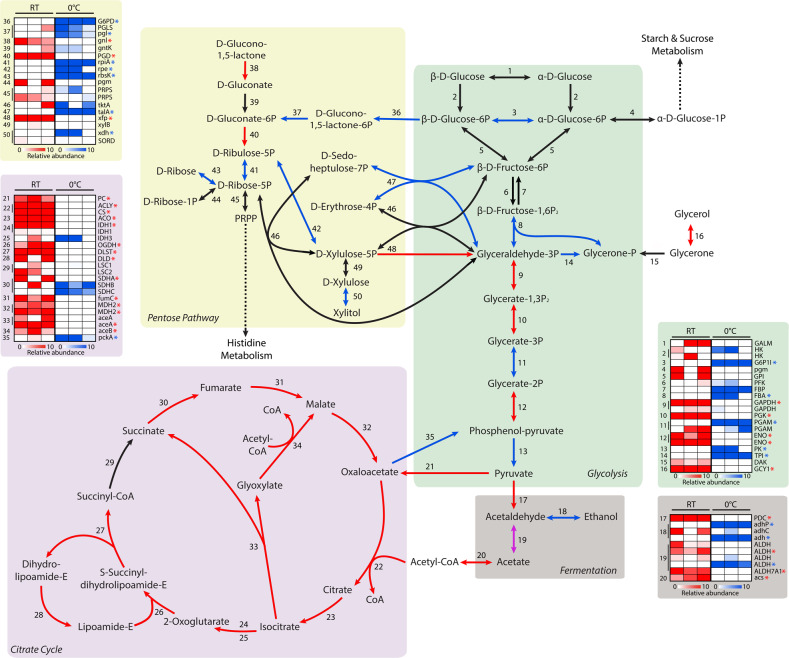
Reconstruction of the *Rhodotorula frigidialcoholis* major carbohydrate metabolic pathways mapped with transcriptomic data. Reconstructed carbohydrate metabolic pathways of *R. frigidialcoholis* based on the KEGG gene annotations and their relative differential gene expression profiles of each triplicate culture. The genes with a significant (*p* < 0.05) differential expression of ≥ 1.5 log_2_FC are indicated with an arrow (pathway) and a star (heatmap) in blue (overexpressed at 0 °C), red (overexpression at 23 °C), or purple (different homolog overexpressed at both 0 and 23 °C). The numbers in the pathways correspond to the numbers in the heatmaps. For the heatmaps, blue indicates an overexpression of the gene at 0 °C, red indicates and overexpression of the gene at 23 °C, and white indicates genes that are not differentially expressed between the two temperatures. A list of the abbreviations is included in the Supplementary material (Supplementary Table S5). .

The majority of carbohydrate metabolism expression differences between 0 and 23 °C were in genes of the citrate cycle, pentose phosphate pathway (PPP), and alcohol fermentation. At 23 °C, *R. frigidialcoholis* significantly overexpressed citrate cycle genes as well as glycolysis genes linked to oxaloacetate and acetyl-CoA syntheses which feed into the citrate cycle (Fig. 4 and Supplementary Table S5). In contrast, at 0 °C, there was a significant increase in the expression of glycolysis genes feeding into ethanol production via fermentation and a significant increase in expression of the PPP genes (Fig. 4).

At 0 °C, *R. frigidialcoholis* overexpressed genes coding for xylulose reductase (FC = 2.40, *p* = 0.00), non-oxidative PPP enzymes, and alcohol dehydrogenase (FC = 8.05, *p* = 0.00), while it downregulated gene coding for 6-phosphogluconate dehydrogenase (FC = 2.21, *p* = 0.00) (Fig. 4). The increase in PPP promotes the production of erythrose-4-phospate, allowing energy (NADH) conservation due to a reduction of the citrate cycle activity [87]. In yeast, including *Rhodotorula*, the PPP is involved in xylulose fermentation [88–90], while downregulation of 6-phosphogluconate dehydrogenase activity is correlated with increased production of ethanol via xylulose fermentation [91]. Xylulose is fermented by xylulose reductase [88] to xylitol, a cryoprotectant and freezing point depressant in yeast [92]. The non-oxidative PPP enzymes can also increase the yield of ethanol fermentation through the conversion of pentose phosphate into intermediates of the glycolysis pathway [90]. A similar upregulation of alcohol dehydrogenase was also observed in the Arctic permafrost bacterium, *Planococcus halocryophilus*, grown at −15 °C [2]. These transcriptional changes in *R. frigidialcoholis* suggest that it adapts to cold temperature by increasing xylitol production and redirecting PPP molecules to ethanol fermentation, which would be beneficial for its survival in extreme cryoenvironments such as Antarctic University Valley.

Antioxidant production through proline-linked PPP was proposed in yeast [93] as an oxidative stress response to cold [87]. *R. frigoalchoholis* can use L-Proline as a sole carbon and nitrogen source (Supplementary Table S2), without reducing the tetrazolium redox dye, suggesting that L-Proline is used by *Rhodotorula* in the proline-linked PPP, leading to decreased citrate cycle activity. This may result in a decrease of the ETC activity and, consequently, the observed lack of tetrazolium dye reduction and a possible redirection of erythrose-4-phosphate to the fermentation. Overall, increased transcription of the PPP genes at cold temperatures could help *R. frigidialcoholis* to conserve NADH conservation, limit carbon loss through CO_2_, and enhance ethanol production to lower the freezing point.

**Fig. 5:**
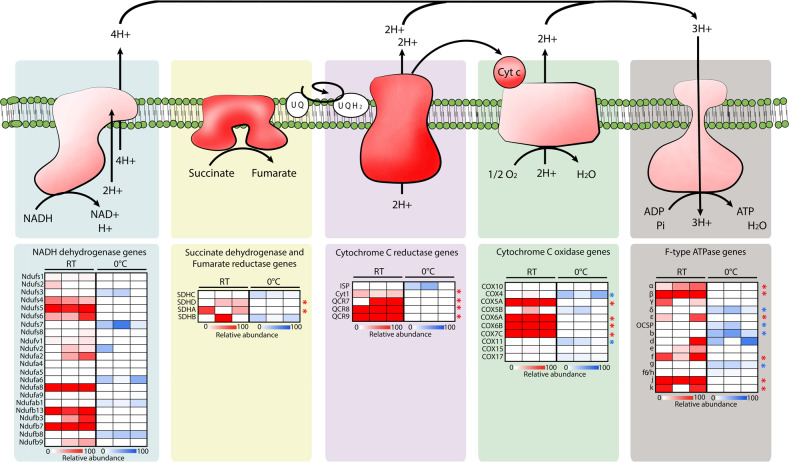
Reconstruction of the *Rhodotorula frigidialcoholis* electron transport chain. Reconstructed electron transport chain (ETC) complexes of *R. frigidialcoholis* based on the KEGG genes annotation (top) and their relative differential gene expression profiles of the ETC subunits (bottom) of each triplicate culture. The genes with a significant (*p* < 0.05) differential expression of ≥1.5 log_2_FC are indicated with a star in blue (overexpressed at 0 °C), or red (overexpression at 23 °C). For the heatmaps, blue indicates an overexpression of the gene at 0 °C, red indicates and overexpression of the gene at 23 °C, and white indicates genes that are not differentially expressed between the two temperatures.

Overall, a higher number of genes related to energy metabolism were downregulated at 0 °C compared to 23 °C (Fig. 2). In accordance with our carbohydrate metabolism results, this significant change was related to an overall downregulation of the genes encoding for the ETC subunits at 0 °C (Fig. 5). The temperature effect on the ETC activity remains unclear, but some studies confirm a general trend in decrease of ETC expression at lower temperatures in microorganisms [8, 94]. The University Valley permafrost bacterium, *Rhodococcus* JG3, also downregulated multiple ETC cytochromes when grown at −5 °C compared to 23 °C [8], and *Pseudomonas putida* downregulated its NADH and ETC succinate dehydrogenase at 10 °C compared to 30 °C [94]. The ETC gene expression patterns in *R. frigidialcoholis* supports our hypothesis related to the lack of dye reduction in the PM assay at 0 °C as being due to the downregulation of the ETC and a switch to fermentative metabolisms to produce energy. This is further supported by the observed switch in gene expression from the citrate cycle pathway at 23 °C to the fermentation ethanol and xylitol pathways at 0 °C for primary energy production (Fig. 4).

### Ethanol production in *Rhodotorula frigidialcoholis*

**Fig. 6:**
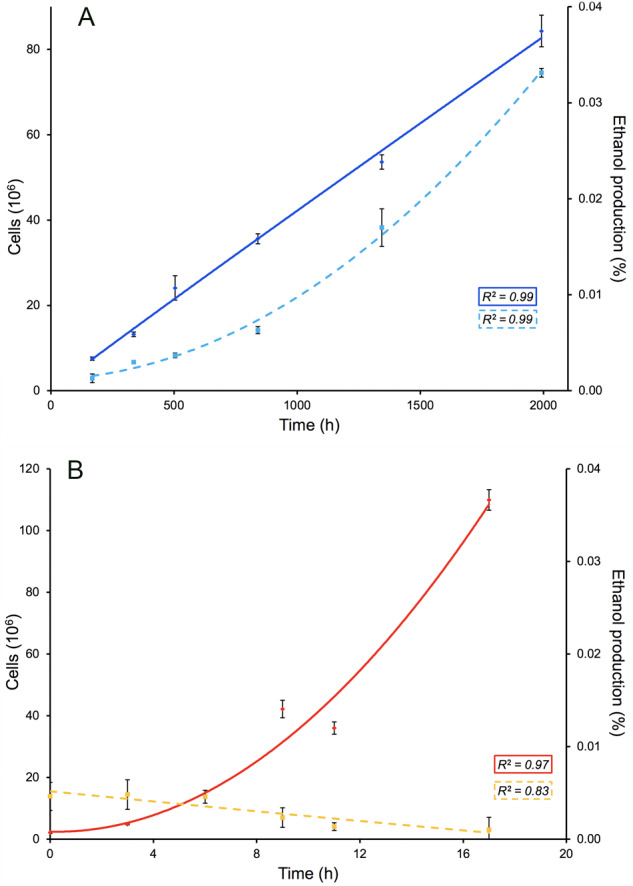
Ethanol production and growth by *Rhodotorula frigidialcoholis* at 0 and 23 °C, in PDB media. **A** Cell growth (diamond, full line) and ethanol production (square, dashed lines) over time at 0 °C. **B** Cell growth (diamond, full line) and ethanol production (square, dashed lines) over time at 23 °C. *R*^2^ values represent the coefficient of determination of the best fit polynomial trendline.

*R. frigidialcoholis’* switch in metabolism from mainly respiratory at 23 °C to fermentative at 0 °C was experimentally confirmed as ethanol production was observed at 0 °C, at an average rate of 1.51 × 10^−5^ moles of ethanol, per liter, per day (Fig. 6A), while ethanol production was not detected at 23 °C (Fig. 6B). Other *Rhodotorula* species, *R. minuta, R. mucilaginosa*, and *R. pallida*, are capable of ethanol fermentation at 28 °C [95]; however, ethanol fermentation at low temperatures in *Rhodotorula* has not been reported. Yeast ethanol production at cold temperatures was reported with *Saccharomyces cerevisiae* in wine (0 and 2 °C) and beer (6 °C) production, although in these studies, fermentation was facilitated by an addition of biocatalysts [96–98]. To our knowledge, *R. frigidialcoholis* is able to naturally produce ethanol at the lowest recorded temperature for microbial fermentation. *R. frigidialcoholis* could then be studied on its potential to isolate cold-temperature active enzymes that promote cellulosic biomass decomposition through fermentation [99], favorizing biofuel production with less energy. Ethanol fermentation has also previously been linked with an increased production of carotenoids in yeast [100], which is consistent with *R. frigidialcoholis* transcriptomic results of increased carotenoid gene expression.

Combined together, downregulation of citrate cycle and ETC genes (Figs 4 and 5), overexpression of fermentation genes (Fig. 4), growth without reduction of the tetrazolium redox dye (Supplementary Table S2), and ethanol production at 0 °C but not at 23 °C suggest that *R. frigidialcoholis* switches from a mainly respiratory metabolism when incubated at 23 °C to a mainly fermentative metabolism when cultured in conditions similar to its natural environment at 0 °C. Ethanol production in *R. frigidialcoholis* may be an ecologically significant adaptive response to subzero temperatures by both acting as a freezing point depressant reducing intracellular freezing and/or to decrease its environment freezing point enhancing the formation of subzero liquid brine vein microhabitats thought to exist within permafrost [101]. Ethanol production could also promote NADH conservation and limiting carbon loss by switching from the citrate cycle to ethanol fermentation. This consequently may increase its survivability in the extremely cold Antarctic permafrost habitat from which it was isolated (Fig. 3).

### Small non-coding RNA expression changes under cold temperature

**Fig. 7:**
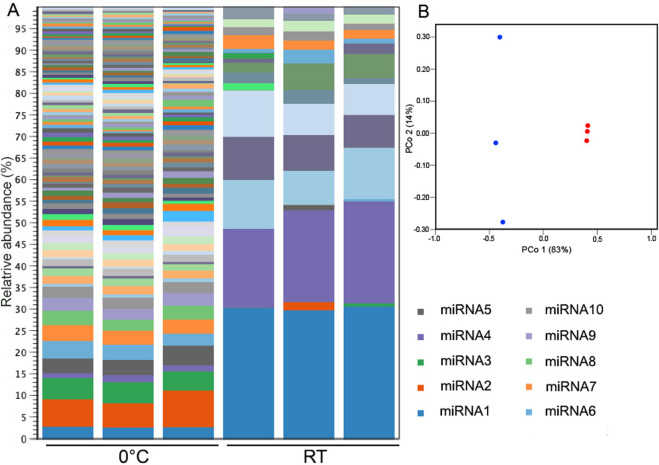
Diversity of short rRFs/miRNAs in *Rhodotorula frigidialcoholis* and PCoA analysis (Bray–Curtis) of *R. frigidialcoholis* short rRFs/miRNAs at 0 and 23 °C. **A** Abundance and diversity of the short rRFs/miRNAs (15–30 nt), with a minimum of 50 reads, of the three 0 °C and the three 23 °C *R. frigidialcoholis* cultures. **B** PCoA analysis (Bray–Curtis) of the three 0 °C (blue) and the 23 °C (red) *R. frigidialcoholis* short rRFs/miRNAs. The legend on the right lists only the ten most abundant short rRFs/miRNAs.

Small non-coding RNA (<200 nucleotides; sRNA) have numerous functions, including protein synthesis (small rRNAs and tRNAs) and mRNA regulation through RNA interference (RNAi) [102]. The roles of sRNA and RNAi are not well understood, but they have been linked to stress response [103, 104]. Based on quantification of the total RNA extracted from *R. frigidialcoholis* cultures, we initially observed a significantly higher (*p* = 0.0003) proportion of sRNAs (<200 nt) in the 0 °C compared to 23 °C cultures (Supplementary Fig. S3). To determine how the sRNA were affected by low temperature in *R*. *frigoalcoholis*, we further analyzed the composition of sRNAs at 0 and 23 °C. On average, 53.40 ± 0.99% of the total RNA was characterized as sRNAs in 0 °C stressed cultures compared to 27.70 ± 1.84% in 23 °C cultures. There are multiple types of sRNA molecules including miRNAs that are non-coding ~22 nt sequences that play a role in gene regulation of eukaryote cells, including fungi [105, 106], and rRFs that are derived from rRNA [107]. We subsequently observed a higher proportion (*p* = 0.029) of short rRFs and miRNA (15–30 nt) in *R. frigoalcoholis* at 0 °C compared to 23 °C (Supplementary Fig. S3). Specifically, 0.69 ± 0.34% of the total RNA was characterized as short rRF/miRNAs in 0 °C stressed cultures compared to 0.02 ± 0.01% at 23 °C, resulting in a ~34-fold increase at 0 °C. In addition, we observed a higher diversity in short rRF/miRNAs at 0 °C compared to 23 °C, based on PCoA analysis (Fig. 7 and Supplementary Fig. S3). Stability and non-random sequence distribution of rRFs in previous studies implies that they are not merely products of rRNA turnover, but a functional part of cellular homeostasis, although they have historically been eliminated from sequencing datasets as contaminants [107]. rRFs can be produced from stressed induced cleavage of tRNAs and rRNAs [107, 108] and globally downregulate translation by binding to ribosomes [109, 110] or by binding to mRNA, both triggering RNAi [107, 111, 112]. Thus, rRF/miRNAs production may be a stress response resulting in overall slower growth rate and metabolism.

Strong links between cold adaptation in prokaryotes and fungi through miRNA regulation have not yet been reported, although this mechanism was identified as a cold adaptation in plants [103]. rRFs have also been linked to cold and UV stress [107], and inhibition of protein translation following DNA damage [113]. Another subset of rRFs are antisense ribosomal small interfering RNAs (risiRNAs) that downregulate pre-rRNA levels through the nuclear RNAi pathway under cold and UV stress to maintain rRNA homeostasis [114, 115]. This phenomenon is triggered by production of erroneous rRNAs transcripts due to environmental stress [116]. Thus, this RNAi pathway potentially decreases global translation by targeting rRNA thus slowing down the cell cycle and limiting production of erroneous proteins.

We could not find any studies clearly reporting the percent of rRFs/miRNAs of total RNA in microorganisms to compare with our results. However, we postulate that the 34-fold increase of rRF/miRNAs at 0 °C in *R. frigidialcoholis* may be a cold temperature response and result from a combination of three possible RNAi mechanisms (Fig. 3): (1) the rRFs were derived from rDNA triggered by DNA damage [113, 115] due to cold stress, resulting in global suppression of mRNA translation, and consequently allowing *R. frigidialcoholis* to slow down its cell cycle under cold temperature; (2) the rRFs observed could act via the nuclear RNAi pathway to downregulate pre-rRNA levels thus suppressing global translation via a negative feedback loop that targets immature rRNA. This pathway may be triggered by an increase in errors during rRNA transcription due to environmental stresses such as low temperature [114]. At 0 °C, we observed rRFs with sequence variability suggesting that erroneous rRNA were potentially produced at a higher rate in *R. frigidialcoholis* at 0 °C than 23 °C. (3) *R. frigidialcoholis* may contain miRNA derived from mRNA and short rRFs derived from rRNA. Both of these could target and suppress a variety of targeted mRNAs in response to cold, as previously observed in plants [103]. Using a RNAhybrid computational method [40], the top putative mRNA targets for the ten most expressed short rRFs/miRNAs (Table 1) were predicted, based on the lowest energy of hybridization required to form a duplex [106]. Of the ten most abundant short rRFs/miRNAs, nine appear to originate from rRNA and one from a hypothetical protein (Table 1). These short rRFs/miRNAs were mainly overexpressed at 0 °C in *R. frigidialcoholis* and potentially target a wide variety of mRNAs as summarized in Table 1. However, we could not directly correlate the increase in expression of the short rRFs/miRNAs with a decrease in expression of their putative mRNA targets, nor do the targets appear to be necessarily linked to cold adaptation. Absence of correlation between specific mRNA target expression levels and short rRFs/miRNAs levels could potentially be due to (a) multiple targets per short rRFs/miRNAs, (b) short rRFs/miRNAs targeting rRNAs (instead of mRNAs) that results in an overall global reduction of translation, or (c) RNAi only partially contributing to gene expression regulation.

**Table 1:** Characteristic of the ten most abundant rRFs/miRNAs expressed in *Rhodotorula frigidialcoholis*.

rRF/miRNA ID	Size (nt)	Origin	Differential expression	Potential targets	Binding energy (kcal/mol)
rRF/miRNA1	30	rRNA	Not differentially expressed	ATPase GET3	−41.6
				Metallo-dependent phosphatase	−38.8
				Protein with homology to sperm nuclear basic protein PL-I isoform PLIb	−38.8
rRF/miRNA2	18	rRNA (28S)	Overexpressed at 0 °C	Glutathione S-transferase	−34.7
				Unknown protein	−34.4
				Unknown protein with acetyl-CoA carboxylase/biotin carboxylase 1 domains	−32.7
rRF/miRNA3	28	rRNA (28S)	Overexpressed at 0 °C	ATP-NAD kinase	−43.2
				Unknown protein with RNA polymerase II degradation factor 1 domain	−41.2
				Ribosome biogenesis protein ERB1	−40.9
rRF/miRNA4	29	rRNA	Overexpressed at 23 °C	ATPase GET3	−41.6
				Protein with homology to sperm nuclear basic protein PL-I isoform PLIb	−39.3
				Ribosome biogenesis protein ERB1	−36.9
rRF/miRNA5	18	rRNA (28S)	Overexpressed at 0 °C	COP9 signalosome complex subunit 7	−34.9
				Mannosyl-oligosaccharide alpha-1,2-mannosidase	−34.5
				RNA polymerase II degradation factor 1	−32.9
rRF/miRNA6	20	rRNA (28S)	Overexpressed at 0 °C	RNA pol II promoter Fmp27 protein domain	−35.9
				NAD + kinase	−35.8
				RNA polymerase II degradation factor 1	−35.3
rRF/miRNA7	22	rRNA	Overexpressed at 0 °C	Staphylococcal nuclease domain-containing protein 1	−33.6
				Unknown protein	−32.8
				Glycogen synthase	−32.7
rRF/miRNA8	22	Unknown protein	Overexpressed at 0 °C	programmed cell death 6-interacting protein	−47.0
				Kinase-like protein	−46.1
				Gutaminyl-tRNA synthetase	−45.6
rRF/miRNA9	29	rRNA (25S)	Overexpressed at 0 °C	Transcription factor IIIB 90 kDa subunit	−51.8
				Unknown protein	−50.9
				Glycosylphosphatidylinositol inositol-deacylase	−50.3
rRF/miRNA10	17	rRNA (28S)	Overexpressed at 0 °C	COP9 signalosome complex subunit 7	−30.5
				26S proteasome regulatory complex component	−30.2
				DHS-like NAD/FAD-binding domain-containing protein	−28.9

Summary of the transcription origin, and differential expression of *R. frigidialcoholis* miRNAs. Putative genomic origin of the rRFs/miRNAs were predicted with BLAST [38] and their putative mRNA gene targets were predicted using the RNAhybrid computational method [40].

Through the action of two RNAse III-type proteins, Drosha and Dicer, miRNAs are produced and recognize specific “target” mRNA that are then silenced via RNAi. Drosha is responsible for the primary transcript cleavage to create precursor miRNA, while Dicer, along with Argonaute (Ago), cleaves the precursor miRNA to form a double strand miRNA. One of these miRNA strands is incorporated into the RNA-induced silencing complex that suppresses translation [106]. Short rRFs have been associated with Ago proteins suggesting that rRFs may act like ribosomal derived miRNA as well [112]. Based on the transcriptomic results, the gene coding for Drosha was not differentially expressed; however, Dicer (FC = 3.83, *p* = 0.01) and RNA polymerase III subunits were significantly overexpressed at 0 °C (Supplementary Table S4). This was consistent with a significantly higher proportion of short rRFs/miRNAs at 0 °C compared to 23 °C and a decrease in the transcript levels of the translational machinery (ribosome proteins and tRNAs) at 0 °C (Fig. 2). Taken together, these results suggest that *R. frigidialcoholis* may induce a short rRFs/miRNAs gene regulatory mechanism in response to cold that triggers translational repression; however, molecular characterizations of the short rRFs/miRNAs, such as stem-loop RT-qPCR and northern blot analysis, are necessary to confirm the involvement of RNAi in cold adaptation.

## Conclusions

Transcriptomic analyses results suggest that *Rhodotorula frigidialcoholis* adapts to cold temperatures in the Antarctic dry valley permafrost through a variety of mechanisms including increasing expression of the PPP genes, increasing the production of carotenoids, sphingolipids, unsaturated fatty acid, and exopolysaccharides while coupled with a reduction in expression of growth, transcriptional and translational machinery genes. We also identified novel cold adaptation features including a switch from respiratory metabolism at 23 °C to fermentative metabolism when grown in a cold condition (0 °C), via downregulation of citrate cycle genes and the ETC genes. In parallel, *R. frigidialcoholis* overexpressed genes involved in ethanol and xylitol fermentation, resulting in ethanol production at the lowest known temperature described so far in any microorganism. This switch may thus be an adaptation to save energy and delay intracellular freezing in cryoenvironments, such as the Antarctic permafrost. At low temperature, *R. frigidialcoholis* also produced a significantly higher proportion of sRNAs, specifically short rRFs/miRNAs, and overexpressed the Dicer gene, suggesting RNAi as a novel mechanism of cold adaptation in polar fungi. Further characterization of the role of ethanol production and identification of mRNA targets of RNAi are needed to determine the roles they play in cold adaptation of yeasts inhabiting the cryosphere. Taken together, our results indicate that *R. frigidialcoholis* has evolved multiple mechanisms to survive in one of the coldest and driest cold environments on Earth.

## Supplementary information

Supplementary File 1

Supplementary File 2

Supplementary File3 Tables and Figures

Supplementary File 4
