# Supplementary material for: Novel Antarctic yeast adapts to cold by switching energy metabolism and increasing small RNA synthesis
Source: ISME J. 2021 Jul 22;16(1):221–32. doi: 10.1038/s41396-021-01030-9 (PMC8692454; doi:10.1038/s41396-021-01030-9)
Supplement: Supplementary file 1 [file 41396_2021_1030_MOESM1_ESM.docx]

**1 Introduction**

The ubiquitous genus of basidiomycetous yeasts *Rhodotorula* (subphylum Pucciniomycotina, class Microbotryomycetes, order Sporidiobolales, family Sporidiobolaceae) was revised in 2015 following the implementation of the “One Fungus = One Name” nomenclatural principle by (Wang *et al* 2015). The revision based on multi-gene phylogenetic analyses comprising nucleotide sequences of the ITS region (including the 5.8S rRNA), the D1/D2 domains of the 28S rRNA, the SSU rRNA, and the RPB1, RPB2, TEF1 and CYTB genes. After being polyphyletic and containing many unrelated species classified as Pucciniomycotina and Ustilaginomycota, the genus currently contains 16 species (Crous *et al* 2017; Wang *et al* 2015), *R. glutinis* being the type species of the genus.

**2 Materials and Methods**

To determine the taxonomy and novelty of *Rhodotorula* JG1b, we performed phylogenetic, phenotypic metabolic, and physiological characterization of this strain. Regions/genes used in the phylogenetic analyses were the small subunit (SSU) rRNA gene, partial large subunit (LSU) rRNA gene including its D1/D2 domain, the internal transcribed spacers 1 and 2 including the 5.8S rRNA gene (ITS), and partial sequences of genes encoding for translation elongation factor 1-α (TEF). These were amplified and sequenced with the following primer sets: NS1/NS24, NL1/NL4, ITS1/ ITS4, EF1-983F/EF1-2218R, respectively (Wang *et al* 2008; White *et al* 1990), as well as whole genome sequences obtained in this study as well as available from public databases. The phylogeny of the concatenated and partitioned alignments of 18S, ITS, 28S rRNA and TEF sequences of representative species within the genus *Rhodotorula* and *Rhodosporidiobolus colostri* used as an outgroup, were reconstructed with MrBayes 3.2.7 (Ronquist *et al* 2012). Two substitution types (allowing for different rates of transitions and transversions) of the 4by4 model and gamma distributed rates with a proportion of invariable sites (approximated with 4 categories of gamma distribution) were used. The estimation was run for 10 million generations (sampling every 100th generation), in 3 runs of 10 chains each, heated at temperature 0.1. The first 25% trees were discarded from the final consensus tree. The genomes were compared to *Rhodotorula* toruloides ATCC 204091 (GCA_000222205) with kSNP3.0 (Gardner *et al* 2015) to produce a VCF file of single nucleotide polymorphisms (SNPs). These were filtered with vcftools (Danecek et al 2011) in order to keep only SNPs on biallelic loci present in at least 75% of compared genomes. There were 55720 loci found that fullfilled these criteria that were used in further anlayses. The dissimilarity distance matrix of the genomes was produced in R with the package ‘poppr’ (Kamvar *et al* 2015) and used for the construction of a neighbour joining tree with the R package ‘ape’ (Paradis and Schliep 2019) using 1000 bootstrap replicates to estimate branch supports.

**3 Results and Discussion**

**Phylogenetic Analysis:**

According to these analyses, the JG1b strain forms a strongly supported monophyletic group within *Rhodotorula* species and is highly supported as the sister clade of *R. mucilaginosa*. Holotype designated herewith: EXF-10854 (Culture Collection of Extremophilic Fungi Ex, University of Ljubljana, Slovenia), permanently preserved in a metabolically inactive state. Ex-type cultures: EXF-10854 and CBS 16468. DNA sequence accession numbers derived from type: MT569975 (18S rRNA), MT569976 (D1/D2 rRNA), MT560678 (ITS), MT584855 (TEF1). MycoBank accession number is MB 835866. Since it is the only representative of the species, no mating reactions could be performed. The novel species is described as forma asexualis (f.a.).

**Detailed description of *Rhodotorula frigidialcoholis***:

After a 14-day incubation at 15 °C on 5% MEA agar, the streaked cultures are slimy, liquid and shinning, slightly lifted with entire margin, greyish red (7B6); colonies reach 9–10 mm in diameter (**Figure 1C**). On PDA, the colonies look similar to those on MEA (**Figure 1A**), reaching 5–7 mm in diameter, reddish orange (7B7). On YPD, streaked cultures look mat, are slightly lifted in centre, convex, with entire margin, from 3–6 mm in diameter; reddish orange (7B8) (**Figure 1B**). Cultures are growing also well on DG18, reaching 3–4 mm in 14 days. The cells are subglobose, budding unipolar (**Figure 1D–F**), (2.5–) 3.5 +/- 0.5 (–4.5) × (3) 4.5+/- 0.5 (-5.5) µm. Cells are encapsulated with up to 0.5 µm thick capsule (**Figure 1F**). Dalmau plate culture on corn meal agar: Pseudohyphae are absent. No sign of glucose fermentation was detected at 5, 15, 20, 30 and 37°C after 12 days of incubation, with the Brom thymol blue and Durham tube technique. A different pattern of nutrient assimilation was observed while comparing strain JG1b to *R. mucilaginosa*, *R. alborubescens* and *R. toruloides* (**Figure S1C**). The following compounds were assimilated: D-glucose, sucrose, raffinose, galactose, trehalose, maltose, melezitose, L-sorbose, L-rhamnose, D-xylose, Dl-arabinose, D-ribose, glycerol, galactitol, D-mannitol, xylitol, L-tartaric Acid, saccharic acid, succinate, D-gluconate. The following compounds were not assimilated: inulin, melibiose, lactose, methyl A-D-glucoside, cellobiose, erythritol, myo-inositol, citrate, D-glucosamine, N-acetyl-D-glucosamine, nitrate, nitrite. A complete list of compounds assimilated and not assimilated by these four species can be found in the **Table S1**. Hydrolysis of urea was positive. The strain JG1b grows at 0, 5, 10, 15, 20, 25, 30, and 37°C (**Figure S4**), but not at 42 °C (data not shown) while incubated in microtiter plates in liquid YNB media. Its growth at marginal temperatures depended on the inoculum size: if too low, no or poor growth was observed. *R.* *frigidialcoholis* was only successfully grown at 0°C while incubated in a PDB 50 ml liquid culture, without shaking.

Several physiological tests allow the differentiation of the two new species from its closest relatives. Contrary to *R. mucilaginosa* and *R. alborubescens*, *R. frigidialcoholis* assimilates L-rhamnose, dulcitol, L-sorbose, and is unable to assimilate citrate and D-cellobiose (**Table S1**).

**4 References**

Crous P, Wingfield M, Burgess T, Hardy GSJ, Barber P, Alvarado P *et al* (2017). Fungal Planet description sheets: 558–624. *Persoonia: Molecular Phylogeny and Evolution of Fungi* **38:** 240.

Danecek P, Auton A, Abecasis G, Albers CA, Banks E, DePristo MA *et al* (2011). The variant call format and VCFtools. *Bioinformatics* **27:** 2156-2158.

Gardner SN, Slezak T, Hall BG (2015). kSNP3. 0: SNP detection and phylogenetic analysis of genomes without genome alignment or reference genome. *Bioinformatics* **31:** 2877-2878.

Kamvar ZN, Brooks JC, Grünwald NJ (2015). Novel R tools for analysis of genome-wide population genetic data with emphasis on clonality. *Frontiers in genetics* **6:** 208.

Paradis E, Schliep K (2019). ape 5.0: an environment for modern phylogenetics and evolutionary analyses in R. *Bioinformatics* **35:** 526-528.

Ronquist F, Teslenko M, Van Der Mark P, Ayres DL, Darling A, Höhna S *et al* (2012). MrBayes 3.2: efficient Bayesian phylogenetic inference and model choice across a large model space. *Systematic biology* **61:** 539-542.

Wang Q-M, Li J, Wang S-A, Bai F-YJAEM (2008). Rapid differentiation of phenotypically similar yeast species by single-strand conformation polymorphism analysis of ribosomal DNA **74:** 2604-2611.

Wang Q-M, Yurkov A, Göker M, Lumbsch H, Leavitt S, Groenewald M *et al* (2015). Phylogenetic classification of yeasts and related taxa within Pucciniomycotina. *Studies in mycology* **81:** 149-189.

White TJ, Bruns T, Lee S, Taylor JJPpagtm, applications (1990). Amplification and direct sequencing of fungal ribosomal RNA genes for phylogenetics **18:** 315-322.
