# Supplementary material for: Novel Antarctic yeast adapts to cold by switching energy metabolism and increasing small RNA synthesis
Source: ISME J. 2021 Jul 22;16(1):221–32. doi: 10.1038/s41396-021-01030-9 (PMC8692454; doi:10.1038/s41396-021-01030-9)
Supplement: Supplementary file 3 — Supplementary File3 Tables and Figures [file 41396_2021_1030_MOESM3_ESM.docx]

**Table S2. OD_590_ values for the Biolog Phenotypic MicroArray at 0°C** OD_590_ values average and normalized both the T0 and the negative control for each “positive” nutrient sources of the PM1 MicroPlate^TM^, PM2A MicroPlate^TM^, and PM3B MicroPlate^TM^ incubated *Rhodotorula* *frigidialcoholis* for 91 days at 0°C. Substrates that caused reduction of the tetrazolium dye are indicated in bold.


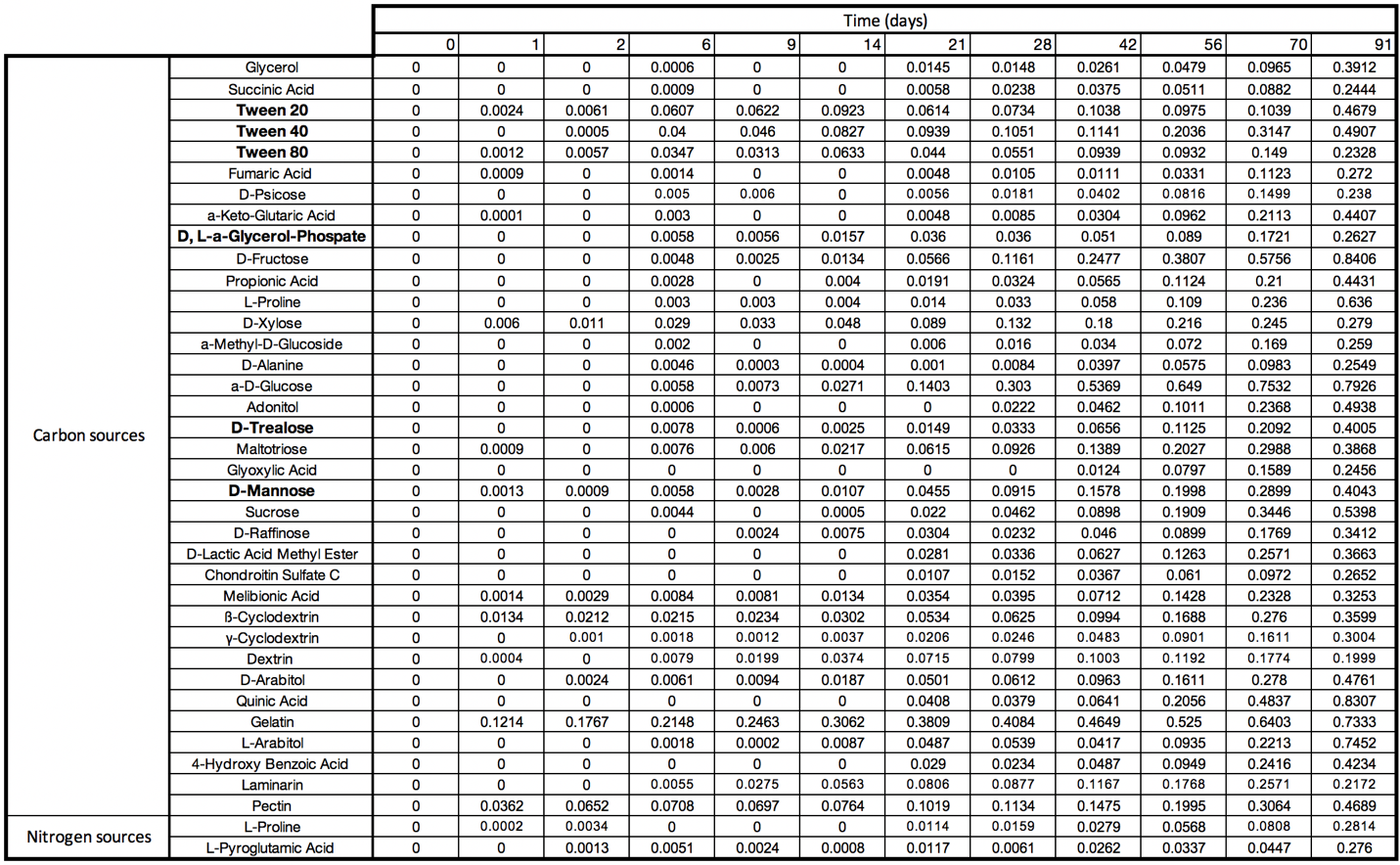


**Table S3. *Rhodotorula* *frigidialcoholis* information and transcriptomic result summary**

|  | ***Rhodotorula* *frigidialcoholis* (Goordial, Raymond-Bouchard et al. 2016)** |
| --- | --- |
| Phylum | Basidiomycota |
| Location isolated | Permafrost,  University Valley, Antarctica |
| Size of genome (Mbp) | 19.3934 |
| GC% | 60.6 |
| Total number of proteins | 6,681 |
| *mRNA transcriptomic results* | (this manuscript) |
| Total number of genes expressed* | 5913 |
| Total differentially expressed genes (≥ 1.5 FC) | 1,772 |
| Increased at 0°C compared to 23°C | 994 |
| Decrease at 0°C compared to 23°C | 778 |
| With KEGG annotation | 924 |
| % KEGG annotation | 52% |

*With an abundance ≥ 1

**Table S5. Figure 3, Figure 4, and Figure S3 list of the abbreviations**

| **Abbreviation** | **Name** | **Abbreviation** | **Name** |
| --- | --- | --- | --- |
| GALM | aldose-1-epimerase | gntK | gluconokinase |
| HK | hexokinase | PGD | 6-phosphogluconate dehydrogenase |
| G6P1I | glucose-6-phosphate 1-epimerase | rpi | ribose 5-phosphate isomerase |
| GPI | glucose-6-phosphate isomerase | rpe | ribulose-phosphate 3-epimerase |
| PFK | 6-phosphofructokinase | rbsK | ribokinase |
| FBP | fructose-1, 6-biphoshatase | PRPS | ribose-phosphate pyrophosphokinase |
| FBA | fructose-bisphosphate aldolase | tkt | transketolase |
| GAPDH | glyceraldehyde 3-phosphate dehydrogenase | tal | transaldolase |
| PGK | phosphoglycerate kinase | xfp | xylulose-5-phosphate/fructose-6-phosphate phosphoketolase |
| PGAM | 2,3-bisphosphoglycerate-dependent phosphoglycerate mutase | xyl | xylulokinase |
| ENO | enolase | xdh | D-xylulose reductase |
| PK | pyruvate kinase | SORD | L-iditol 2-dehydrogenase |
| TPI | triosephosphate isomerase | SPT | serine palmitoyltranferase |
| DAK | triose/dihydroxyacetone kinase | KDSR | 3-keto-deihydrosphingosine reductase |
| GCY1 | glycerol 2-dehydrogenase | LAG | ceramide synthase |
| PDC | pyruvate decarboxylase | DEGS | sphingolipid 4-desaturase/C4-monooxygenase |
| Adh | alcohol dehydrogenase | SMPD | sphingomyelin phosphodiesterase |
| ALDH | aldehyde dehydrogenase | UGCG | ceramide glucosyltransferase |
| Acs | acetyl-CoA synthetase | SUR | sphinganine C4-monooxygenase |
| PC | pyruvate carboxylase | LCB3 | dihydrosphingosine 1-phosphate phosphatase |
| ACLY | ATP citrate lyase | SPHK | sphingosine kinase |
| CS | citrate synthase | SGPL1 | sphinganine-1-phosphate aldolase |
| ACO | aconitate hydratase | Biosyn | biosynthesis |
| IDH | isocitrate dehydrogenase | FA | fatty acids |
| OGDH | 2-oxoglutarate dehydrogenase | syn | synthesis |
| DLST | dihydrolipoamide succinyltransferase | EPS | extracellular polysaccharide |
| DLD | dihydrolipoamide dehydrogenase | RNA PIII | RNA polymerase III |
| LSC | succinyl-CoA synthetase | QDC-3-like | protein required to produce RNA molecules form damaged DNA |
| SDH | succinate dehydrogenase | RDRPs | RNA-dependant RNA polymerase |
| fum | fumarate hydratase | sRNA | short non-coding RNA |
| MDH | malate dehydrogenase | rRFs | rRNA fragments |
| ace | isocitrate lyase | miRNA | microRNA |
| pck | malate synthase | PP | pentose phosphate |
| G6PD | glucose-6-phosphate 1-dehydrogenase |  |  |
| PGLS, pgl | 6-phosphogluconolactonase |  |  |
| gnl | gluconolactonase |  |  |

**Table S6. *Rhodotorula* *frigidialcoholis* differentially expressed homologous proteins summary**

| **KO Number** | **KO  Name** | | **No. of homologs/analogs equally expressed** | | **No. of homologs/analogs overexpressed at 23°C** | | | **No. of homologs/analogs overexpressed at 0°C** | | **Function** | | |
| --- | --- | --- | --- | --- | --- | --- | --- | --- | --- | --- | --- | --- |
| **K00101** | lldD | | 0 | | 2 | | | 1 | | L-lactate dehydrogenase (cytochrome) | | |
| **K00128** | ALDH | | 2 | | 1 | | | 1 | | aldehyde dehydrogenase (NAD+) | | |
| **K00698** | CHS1 | | 3 | | 2 | | | 1 | | chitin synthase | | |
| **K01183** | - | | 2 | | 1 | | | 1 | | chitinase | | |
| **K01379** | CTSD | | 2 | | 1 | | | 2 | | cathepsin D (aspartic endo-protease) | | |
| **K01273** | DPEP | | 0 | | 1 | | | 1 | | * membrane dipeptidase | | |
| **K01530** | - | | 0 | | 1 | | | 1 | | * phospholipid-translocating ATPase | | |
| **K03457** | TC.NCS1 | | 1 | | 1 | | | 1 | | * nucleobase:cation symporter-1, NCS1family | | |
| **K06689** | UBE2C | | 1 | | 1 | | | 1 | | ubiquitin-conjugating enzyme E2 D | | |
| **K10756** | RFC3_5 | | 0 | | 1 | | | 1 | | replication factor C subunit 3/5 | | |
| **K11253** | H3 | | 2 | | 3 | | | 1 | | histone H3 | | |
| **K13348** | MPV17 | | 0 | | 1 | | | 2 | | *protein Mpv17 | | |
| **K14686** | SLC31A1 | | 0 | | 1 | | | 1 | | *SLC31A1, CTR1; solute carrier family 31 (copper transporter), member 1 | | |
| **K15109** | SLC25A20_29 | | 3 | | 1 | | | 1 | | * solute carrier family 25 (mitochondrial carnitine/acylcarnitine transporter), member 20/29 | | |
| **K16261** | YAT | | 2 | | 2 | | | 3 | | * yeast amino acid transporter | | |
| **K18065** | CDC25 | | 0 | | 1 | | | 1 | | Cdc25 family phosphatase | | |
| **K19355** | MAN | | 1 | | 1 | | | 1 | | mannan endo-1,4-beta-mannosidase | | |
| **K21989** | TMEM63 | | 1 | | 1 | | | 1 | | * calcium permeable stress-gated cation channel | | |
| **Total** |  | | 20 | | 23 | | | 22 | |  | | |
|  | |  | |  | | |  | | | |  |  |
| * Genes involved with cellular membranes | | | | | |  | | |  | | |  |

**
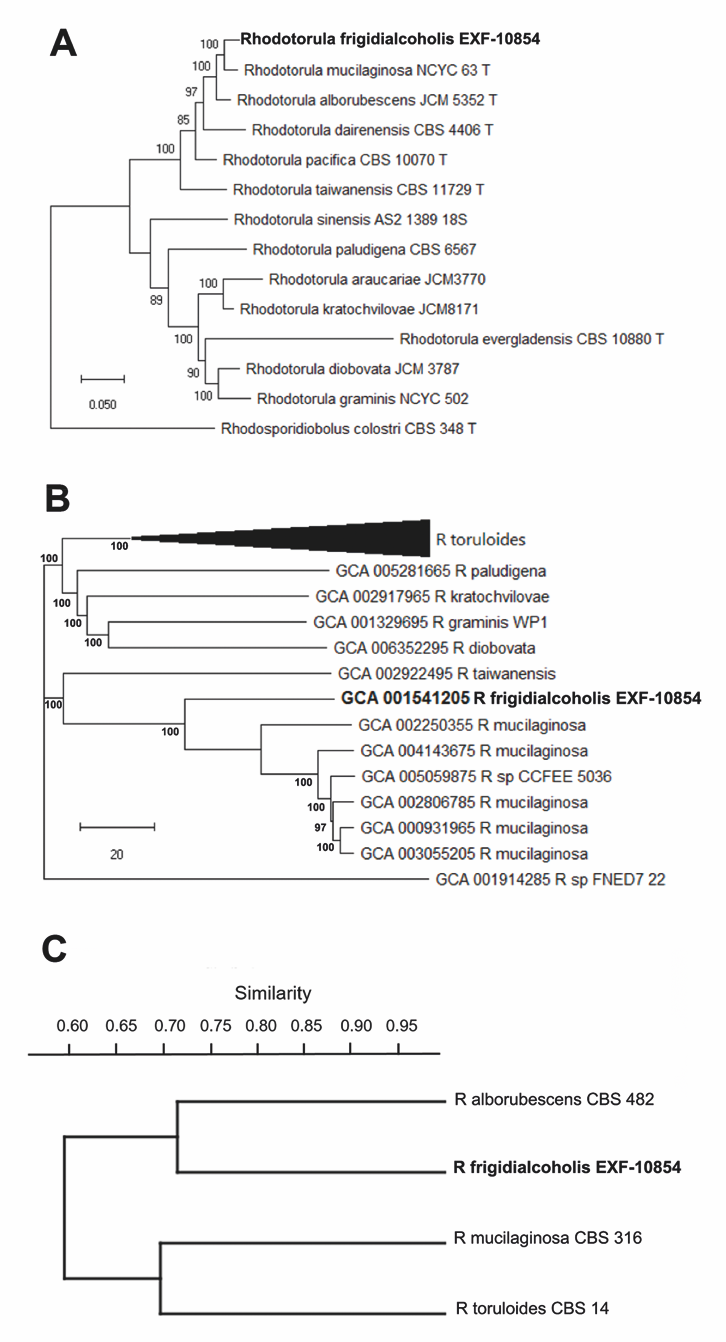
**

**Figure S1. Phylogenetic and phenotypic characterization of *Rhodotorula frigidialcoholis***

(**A**) Phylogenetic tree estimated by Bayesian inference from alignment of the 18S, ITS, 28S rRNA and TEF sequences. 10 million generations were calculated, the trees were sampled every 100 generations. The shown consensus tree was calculated from all sampled trees except the first 25%. The numbers given on branches are frequencies at which the individual clades appeared in the sampled trees. (**B**) Neighbor-joining tree calculated from distances between the genomes calculated from single-nucleotide polymorphism data as estimated by kSNP3. (**C**) Cladogram drawn based on hierarchical clustering of Biolog’s carbon and nitrogen assimilation assays data using Jaccard similarity index.

**Figure S2. Growth curves of *Rhodotorula* *frigidialcoholis*, *R. mucilaginosa* CBS 316, and *R. toruloides* CBS 14 in microtiter plates in YNB media, at different temperatures.**


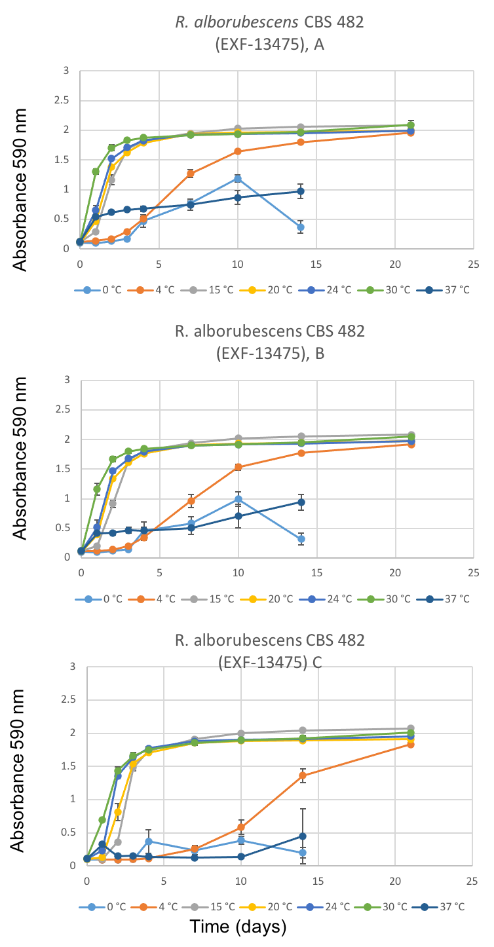

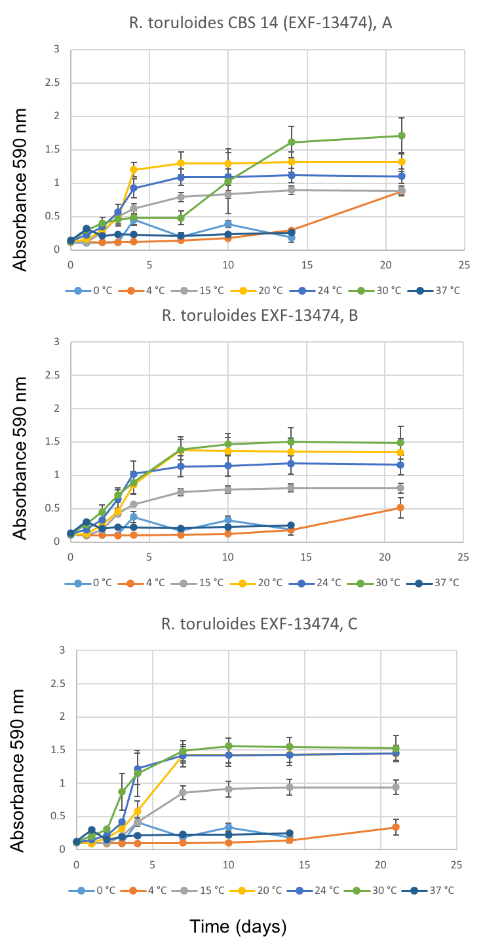

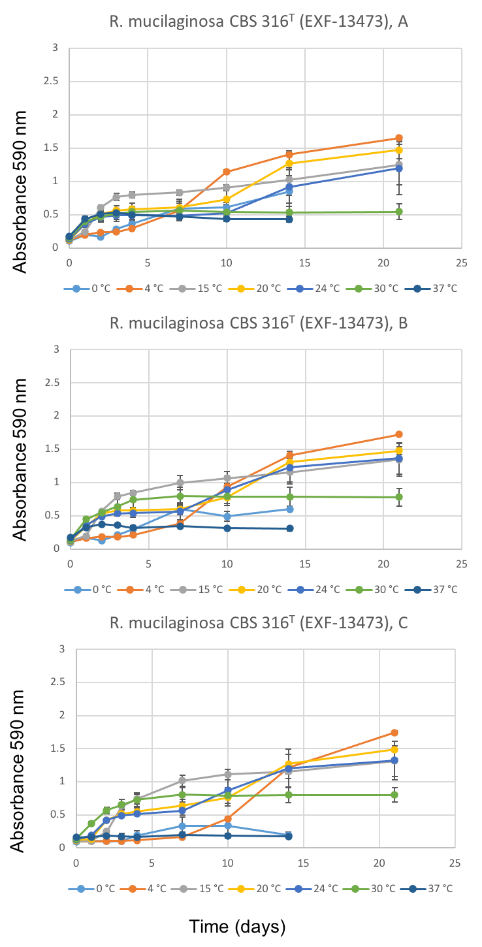

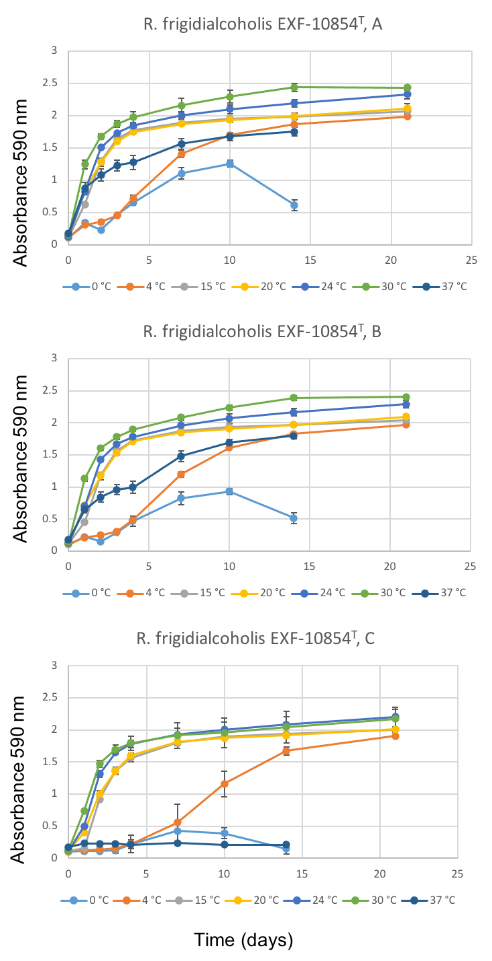

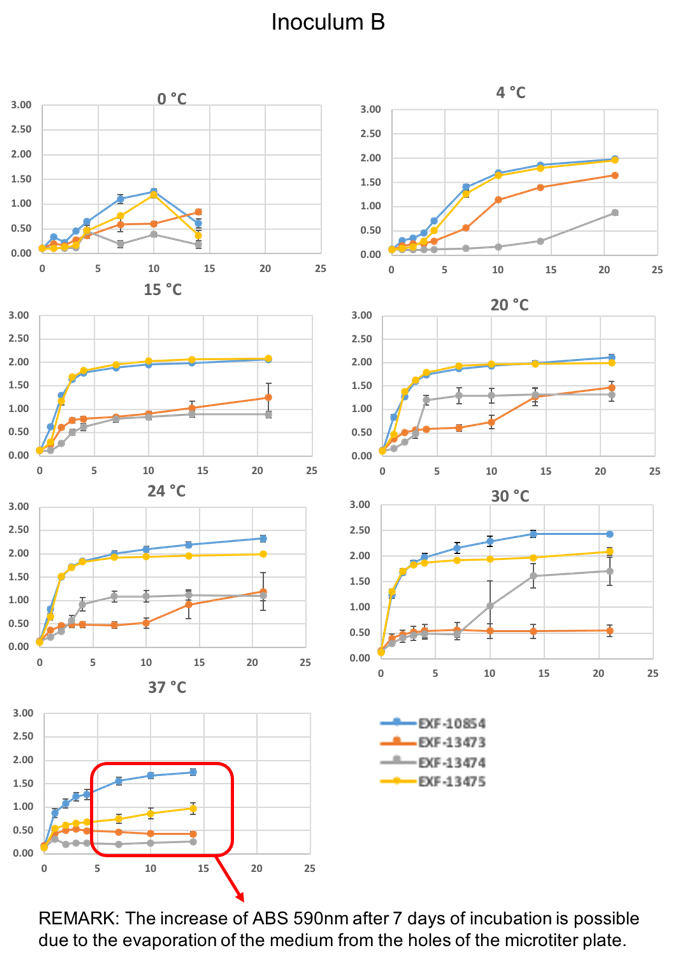


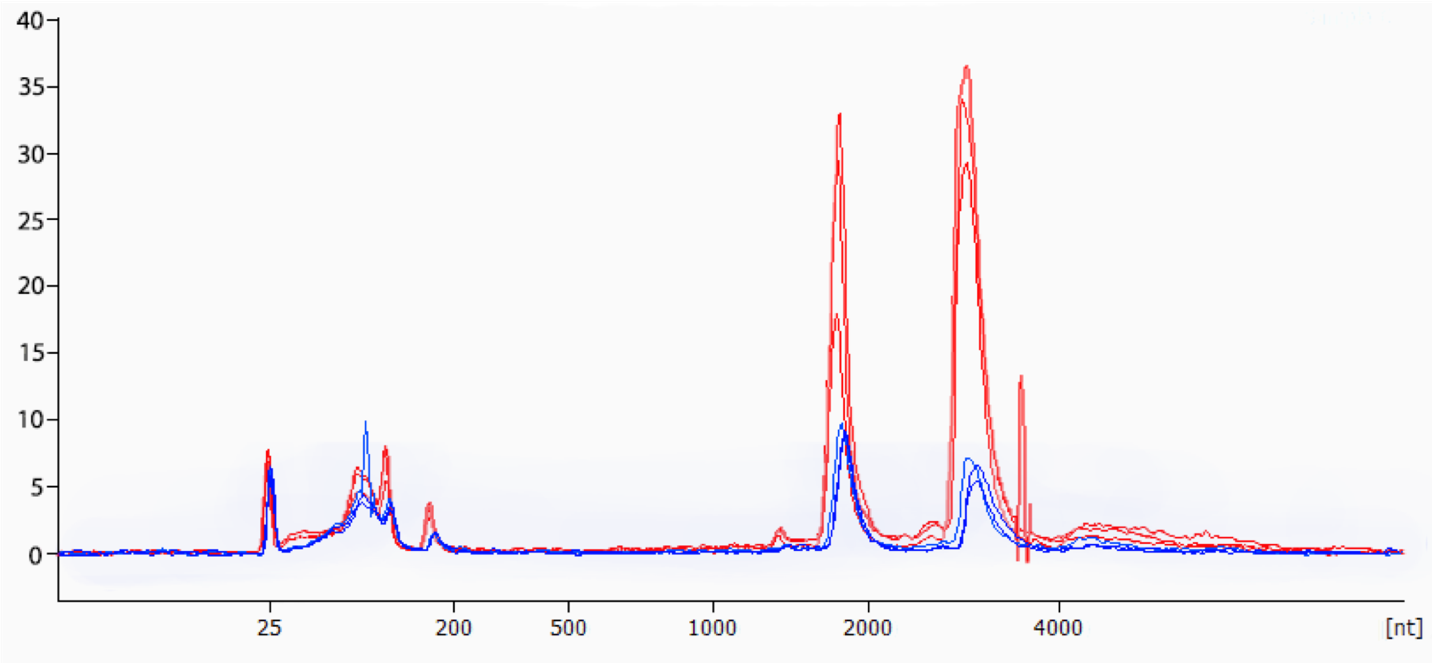


**A**

**B**

**
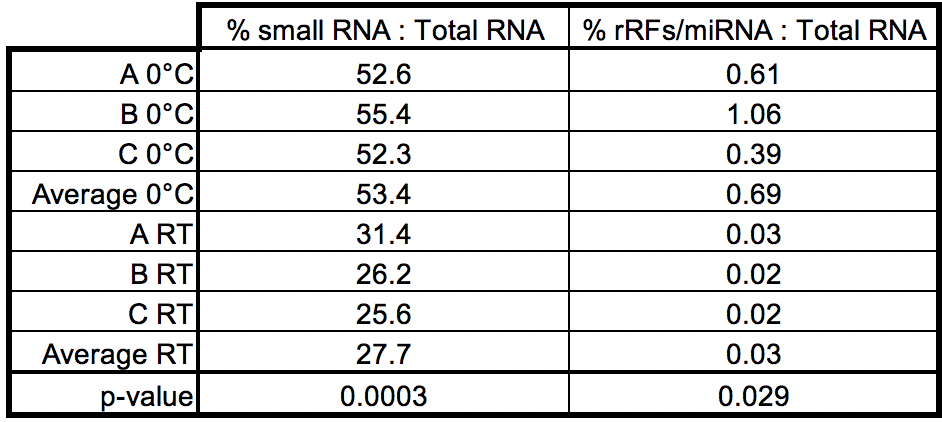
**

**Figure S3. Proportion of Total RNA, small RNA and rRFs/miRNA for the *Rhodotorula* *frigidialcoholis* at 0°C and 23°C.**

(**A**) Superposition of the Total RNA 2100 Bioanalyzer system electrogram extracted from the three 23°C *Rhodotorula* *frigidialcoholis* cultures (*red*), and the three 0°C *Rhodotorula* *frigidialcoholis* cultures (*blue*). (**B**) Summary of small RNA and miRNA proportion in *Rhodotorula* *frigidialcoholis* cultures grown at 0°C and 23°C, determined based on the 2100 Bioanalyzer system results.


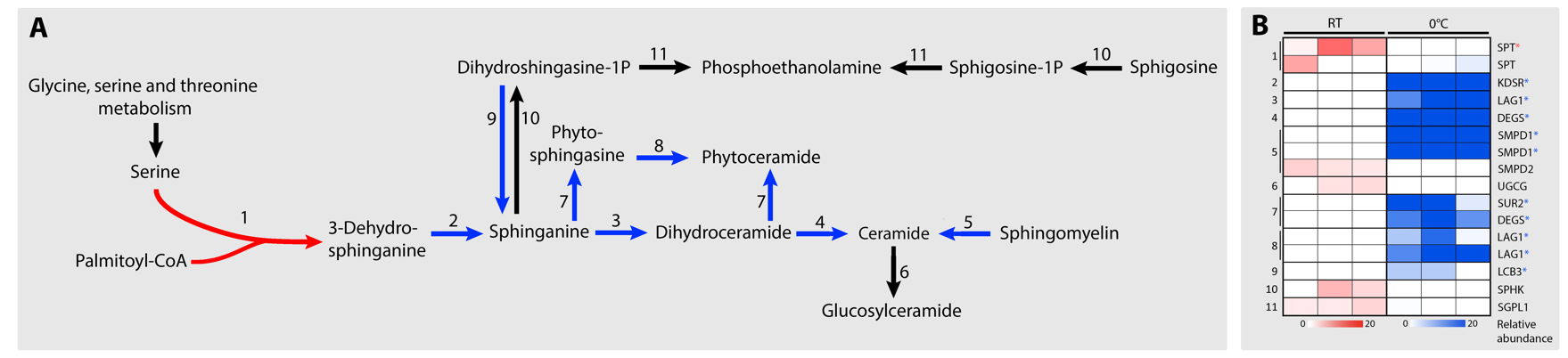
**Figure S4. Reconstruction of the *Rhodotorula frigidialcoholis* sphingolipid metabolic pathways mapped with transcriptomic data**

(**A**) Reconstructed sphingolipid metabolic pathways of *R. frigidialcoholis* based on the KEGG genes annotation. (**B**) Differential gene expression profiles of the sphingolipid metabolic pathway genes of each triplicate culture. The genes with a significant (p < 0.05) differential expression of ≥ 1.5 log_2_FC are indicated with an arrow (**A**) and a star (**B**) in *blue* (overexpressed at 0°C), or *red* (overexpression at 23°C). The *numbers* in the pathways correspond to the numbers in the heatmaps. For the heatmaps, *blue* indicates an overexpression of the gene at 0°C, *red* indicates and overexpression of the gene at 23°C, and *white* indicates genes that are not differentially expressed between the two temperatures. A list of the abbreviations is included in the supplemental material (**Table S6**).
